# Effectiveness of solution-focused brief therapy on cancer patients: a systematic review and meta-analysis

**DOI:** 10.3389/fpsyg.2026.1741088

**Published:** 2026-02-12

**Authors:** Luqiang Zhou, Xiaojuan Yuan, Yan Li, Siyue Li, Fang Guo, Caiping Song, Yu Chen, Xi Xiong

**Affiliations:** 1 Department of Obstetrics and Gynecology, Second Affiliated Hospital, Army Medical University, Chongqing, China; 2 Department of Urology, Urologic Surgery Center, Second Affiliated Hospital, Army Medical University, Chongqing, China; 3 Department of Office of the Hospital, Second Affiliated Hospital, Army Medical University, Chongqing, China

**Keywords:** anxiety, cancer, cancer-related fatigue, depression, quality of life, SFBT, solution focused brief therapy

## Abstract

**Aim:**

The aim of this study was to review and meta-analyze the effectiveness of a nursing intervention based on solution-focused brief therapy (SFBT) in improving anxiety, depression, cancer-related fatigue, and quality of life in cancer patients.

**Methods:**

Conducting a systematic evaluation and meta-analysis following the PRISMA guidelines, we performed a thorough search across various databases including Cochrane, PubMed, CINAHL, PsycInfo, EMBASE, CNKI, Wanfang Database, and VIP Database. The search was conducted from the inception of each database up to February 2024. The overall effect size of the intervention was determined by calculating the standardized mean difference (SMD) and its 95% confidence interval. Statistical analyses were conducted using Review Manager (RevMan) 5.4.1.

**Results:**

13 studies meeting the selection criteria were included, encompassing a total of 1146 patients in the final analysis. 8 research examined the impact on anxiety, 10 on depression, 6 on cancer-related fatigue, and 5 on quality of life. The standardized mean difference for anxiety was –1.52 (CI: –2.20 ∼ –0.84, *p* < 0.0001), depression was –1.54 (CI: –2.09 ∼ –1.00, *p* < 0.00001), cancer-related fatigue was –2.19 (CI: –3.33 ∼ –1.05, *p* = 0.0002), and quality of life status was 2.18 (CI: 0.73 ∼ 3.62, *p* = 0.003). The overall certainty of the evidence was rated low due to limitations such as lack of allocation concealment, blinding, lack of clinical trial registration for the majority of studies, and high risk of bias in several areas.

**Conclusion:**

SFBT may be effective in reducing anxiety, depression, cancer-related fatigue and improving quality of life in cancer patients. However, due to the limitations of the original study, including a high risk of bias and significant heterogeneity, although the research results are encouraging, the overall quality of existing evidence is low, so we cannot draw clear conclusions. More robust research designs are needed to accurately evaluate the effects of this treatment.

## 1 Introduction

Cancer remains a major global public health challenge, with nearly 10 million deaths annually worldwide (Saudi Medical Journal, 2024). In the United States, projections for 2025 indicate over 2 million new cases and approximately 618,000 deaths (Siegel et al., 2025). China reports 4.29 million new cases annually, representing 20% of the global incidence, with 2.81 million deaths (Han et al., 2024). Beyond physical morbidity, cancer patients frequently experience significant psychological distress, including fear, treatment skepticism, and concerns about family and future (Tang et al., 2024), often stemming from disease uncertainty, lack of social support, and existential threat appraisals (Castelli et al., 2015). Meta-analytic data indicate a psychological distress prevalence of ∼53.7% among cancer patients, with depression and anxiety affecting about 24.6 and 10.3%, respectively (Mitchell et al., 2011), though prevalence ranges widely (e.g., depression 21∼77.9%; anxiety 18.5∼40%) (Zeilinger et al., 2022). Cancer-Related Fatigue (CRF) is a pervasive and distressing symptom, characterized by persistent, subjective tiredness unrelated to activity and unrelieved by rest (Vardhan et al., 2022). Its multifactorial etiology involves cytokine dysregulation, metabolic disturbances, anemia, pain, and psychological stress (Agbejule et al., 2022). CRF encompasses physical, emotional, and cognitive dimensions, such as low mood and concentration difficulties. With no specific pharmacotherapy, management relies on comprehensive interventions including physical activity, nutrition, and psychological support (Shambhavi and Lobo, 2021). Therefore, exploring effective psychological interventions to minimize CRF is of great importance in enhancing physical and mental well-being of patients. Quality of Life (QoL) is a key outcome in cancer care, encompassing physical, psychological, social, and spiritual domains (De Souza et al., 2024). Symptoms like pain, fatigue, and nausea impair physical function and comfort, while psychological distress and social role changes affect mental and social adjustment (Chui, 2019; De Souza et al., 2024). Fatigue and pain notably disrupt daily activities, work, and social interactions, thereby reducing QoL (Ferreira et al., 2008; Salvetti et al., 2020). Depression and anxiety further contribute to QoL decline. Major oncology societies emphasize integrating QoL assessment and improvement throughout cancer treatment (Andersen et al., 2014; Grassi et al., 2023).

Solution-focused brief therapy (SFBT), rooted in constructivism, emphasizes resource-oriented cognition and solution-building through techniques like exceptional questioning and scaling (Franklin et al., 2017). It is a goal-directed modality that prioritizes solutions over problem analysis (Harshita and Rubina, 2022). In oncology, SFBT may alleviate psychological symptoms by boosting self-efficacy, facilitating meaning-making, and enhancing coping flexibility. Its positive, time-efficient approach is particularly suitable for cancer patients, who often have limited energy and need rapid support. A pilot RCT by Zhang et al. demonstrated SFBT reduced psychological distress and increased hope in adolescent and young adult cancer patients (Zhang et al., 2022). Other studies report benefits for sexual quality of life (Bokaie et al., 2023) and overall QoL (Man et al., 2019), alongside improvements in anxiety, depression, and CRF across various cancer types.

In summary, existing evidence suggests SFBT may positively impact anxiety, depression, CRF, and QoL in cancer patients. However, heterogeneity in samples, interventions, and outcomes across studies precludes definitive conclusions. This study aims to systematically evaluate the efficacy of SFBT on these outcomes via meta-analysis, providing an evidence base for clinical practice and further research.

## 2 Materials and methods

### 2.1 Protocol

The Cochrane Collaboration’s suggested framework was used in the creation of this review. The Preferred Reporting Items for Systematic Reviews and Meta-Analyses (PRISMA) statement was followed in the reporting of all outcomes. The protocol of this study was registered with PROSPERO (reg. no. CRD42024560622) (https://www.crd.york.ac.uk/PROSPERO/view/CRD42024560622).

### 2.2 Search strategy

In February 2024, we conducted a literature search on Cochrane, PubMed, CINAHL, PsycInfo, EMBASE, China Biomedical Literature Database, CNKI, Wanfang Database, and VIP Database to identify studies examining the treatment outcomes and effectiveness of SFBT in cancer patients. The search time starts with the building of the library and the results are not limited by language.

The Cochrane Collaborative Network’s optimal sensitivity technique was applied to find RCTs in PubMed. A similar term-based search method was used to find and identifying studies of SFBT in treatment outcomes in cancer patients. Sensitive searches were performed using keywords and their synonyms, and free-text retrieval was carried out with terms like “tumor/cancer/hematologic neoplasms/lymphoma/myeloma/leukemia” and “focal solution treatment/focal solution/Solution-Focused Brief Therapy/Solution-Focused Therapy/Solution-Focused/SFBT.” Table 1 provides an example of how to use the search technique in PubMed.

**TABLE 1:** In the case of PubMed, for example.

Search strategy
#1 tumor OR cancer OR hematologic neoplasms OR lymphoma OR myeloma OR leukemia
#2 focal solution treatment OR focal solution OR Solution-Focused Brief Therapy OR Solution-Focused Therapy OR Solution-Focused OR SFBT
#3 #1 AND #2

Each identified abstract was independently reviewed by two authors (YL and SyL). If at least one of the writers deemed a reference eligible, the complete text was retrieved for a thorough review. They independently evaluated the entire text of the selected articles to ensure they met the criteria for inclusion or exclusion. In case of any disagreement, the writers addressed the reasons for their decisions, and a consensus was achieved.

### 2.3 Inclusion and exclusion criteria

We conducted a comprehensive search and analysis of all available studies on the application of SFBT in cancer patients. Our search encompassed published papers, gray literature, and materials acquired through additional manual searches of the literature.

The included randomized controlled studies evaluated the use of SFBT as an intervention to assess psychological distress and/or CRF and QoL in cancer patients over the age of 18. Studies were excluded if they (a) lacked original patient data, (b) was an editorial comment, a letter, a review, a case report, an author’s reply, (c) was not in Chinese and English, (d) Chinese articles published in non-Chinese science and technology core journals (this standard aims to prioritize research that has undergone more mature, internationally recognized peer review processes, while acknowledging that this approach may result in the exclusion of some studies with local relevance), (e) articles in Chinese with only one author (clinical studies cannot be done by one person), or (f) were unpublished studies with only abstracts presented at national and international conferences. The references of all listed publications were searched to find more relevant studies.

### 2.4 Data extraction

Two authors (FG and XjY) extracted descriptive and outcome data from the included studies using pre-established standardized forms. We considered the following aspects: (a) characteristics of the investigated, including name, nationality, and publication date; (b) details of the intervention conducted, such as tumor type, age, sample size, and study content; (c) SFBT technique; (d) lost to follow-up (if applicable); (e) evaluation indicators; and (f) presentation of results. If duplicate cohorts were suspected to be from the same author or institution, higher quality or more recent data were used in the analyses. All discrepancies related to data extraction were resolved through consensus with co-authors.

### 2.5 Risk of bias assessment

All studies included in the research (regardless of language or journal) underwent a standardized, rigorous risk of bias assessment, ensuring transparency in the quality evaluation. The risk of bias of the included studies was assessed using the risk of bias assessment tool recommended by the Cochrane Review’s Handbook (5.1.0), which consists of seven items, and the judgment of “low risk,” “unclear,” and “high risk” was made for each item. The risk of bias assessment was also done independently by two researchers (YL and SyL), and their assessments were agreed upon through comparison and discussion, with a third person (LqZ) acting as a mediator to deal with disagreements.

### 2.6 Quality appraisal of evidence

To assess evidence quality, two evaluators (YL and SyL) applied the GRADEprofiler to determine the quality of evidence for all outcomes. Evidence quality was classified into four levels: “very low,” “low,” “moderate,” and “high.” Although evidence from randomized controlled trials was initially rated as “high” quality, it was downgraded due to the following five factors: study limitations (risk of bias), inconsistency of results, indirect evidence, imprecision of results, and reporting bias.

### 2.7 Statistical analyses

Meta-analyses were performed using RevMan 5.4.1. The outcome indicators included in the literature for this study were continuous variables, and as different assessment tools were chosen for different studies, the standardized mean difference (SMD) was selected as an indicator of effect size, and a 95% confidence interval (CI) value was calculated to assess the effectiveness of the intervention. SMD in contrast the difference scores before and after intervention between the intervention team and the control group. Heterogeneity of research questions was tested by Cochran’s Q and *I*^2^. *p* ≥ 0.1 and *I*^2^ ≤ 50% is considered low heterogeneity and analyzed using a fixed-effects model; *p* < 0.1 and *I*^2^ > 50% is considered high heterogeneity and analyzed using a random-effects model. We used the “calculator” in the “RevMan” software to calculate the combined SE value and then calculated the SD value using the formula $\mathrm{SD}=\text{SE}\times \sqrt{\text{N}}$.

For subgroup analyses based on SFBT intervention duration (short-term effect: within 3 months, medium-term effect: 3–6 months), country, assessment scale, and tumor type were performed to explore heterogeneity between different subgroups. Sensitivity analyses through case-by-case exclusion.

## 3 Results

### 3.1 Sample of included studies

**FIGURE 1:**
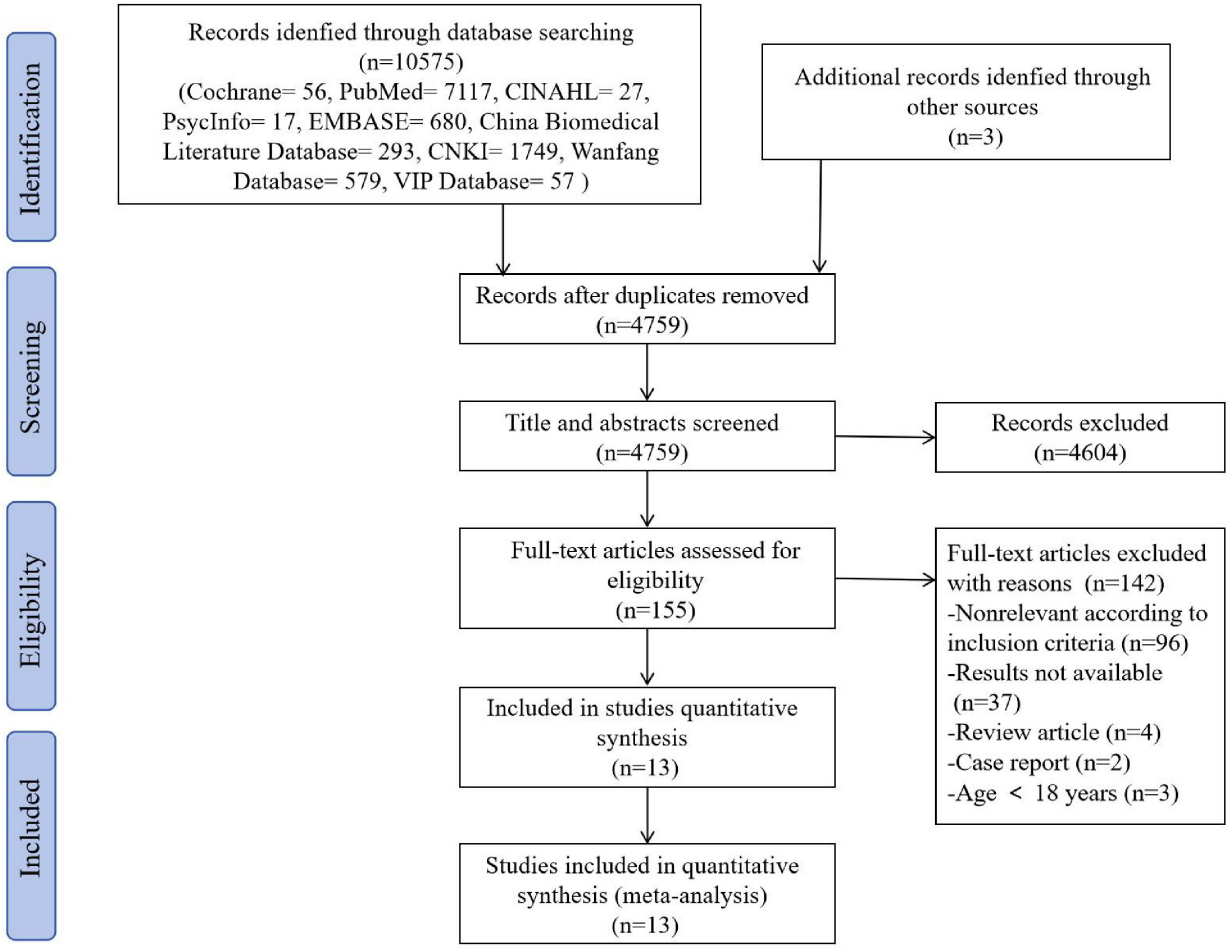
Search and selection of studies for systematic review according PRISMA.

A total of 10,575 articles were obtained through the initial search, 3 articles were obtained through other means, and 4,759 articles remained after de-duplication and management using Endnote 9.0. After screening by reading the titles and abstracts, 155 articles were retained, while 142 articles were excluded after further in-depth reading of the articles, and the final 13 articles included in this study. See Figure 1 for a detailed description of the inclusion and exclusion process according to PRISMA.

All eligible studies were published between 2014 and 2023. Among them, there are six studies in English (Aminnasab et al., 2018; Liu et al., 2020; Man et al., 2019; Pirzadi et al., 2023; Wang et al., 2021; Xian et al., 2022) and 7 studies in Chinese (Huiqin et al., 2015; Jia et al., 2023; Jie and Weilian, 2016; Jie et al., 2014; Mei et al., 2019; Qianqian et al., 2023; Xiaoqun et al., 2016). Two studies were from Iran (Aminnasab et al., 2018; Pirzadi et al., 2023) and the rest were from China (Huiqin et al., 2015; Jia et al., 2023; Jie and Weilian, 2016; Jie et al., 2014; Liu et al., 2020; Man et al., 2019; Mei et al., 2019; Qianqian et al., 2023; Wang et al., 2021; Xian et al., 2022; Xiaoqun et al., 2016). Only one study (Aminnasab et al., 2018) explicitly stated follow-up data, which was not meta-analyzed given that none of the other studies had follow-up data. Three studies (Jie et al., 2014; Liu et al., 2020; Xian et al., 2022) reported that participants were lost to visits due to withdrawal, transfer to hospital, deterioration of condition, or off-site. The total sample size of the included studies was 1,146 cases, with 576 cases in the intervention group and 570 cases in the control group. The demographics of each included study are summarized in Table 2.

**TABLE 2:** Demographics of included studies.

Study	Country	Results	Type of cancer	Mean age (exp vs. con)		Sample (exp vs. con)	Duration of intervention	SFBT technique	Dropout	Evaluation
Aminnasab et al. (2018)	Iran	+	Breast Cancer	20–45		15 vs. 15	2 h of psychotherapy once a week,8 times, 1 mouth follow up	➀ ➁ ➂ ➃ ➄	Not reported	PSS, CES-D
Xian et al. (2022)	China	+	Colorectal Cancer	59.12 ± 11.36	62.63 ± 9.46	60 vs. 59	30 min on the first day of chemotherapy, once a month for 6 months,1 vs. 1	➀ ➁ ➂ ➃ ➄	7 transfers, 7 discontinued withdrawals, 5 lost visits, 1 withdrawal due to off-site location	CFS-C,QLICP-CR
Man et al. (2019)	China	+	Cervical Cancer	50.52 ± 3.16	50.38 ± 3.28	45 vs. 45	SFBT prior to surgery, hospitalization	➀ ➁ ➂ ➃ ➄	Not reported	SAS;SDS,GQOL-74
Pirzadi et al. (2023)	Iran	+	Breast cancer	38.32 ± 8.73	38.89 ± 8.04	28 vs. 28	1 time per week, one 90-min session, for a total of 6 sessions	➀ ➁ ➂ ➃ ➄	Not reported	BDI,WHOQOL- BREF
Qianqian et al. (2023)	China	+	Bladder cancer	66.96 ± 4.55	67.45 ± 4.55	40 vs. 40	Every 2 weeks, 4 times for a total of 8 weeks	➀ ➁ ➂ ➃ ➄	Not reported	kps,SIS,SAS,SDS
Liu et al. (2020)	China	+	Breast cancer	40–60		66 vs. 67	8 weeks, 1 h per week, 12 weeks follow up	➀ ➁ ➂ ➃ ➄	23 lost visits for unspecified reasons	PFS
Wang et al. (2021)	China	+	Leukemia	42.28 ± 1.09	42.32 ± 1.02	51 vs. 51	1 time per week for 20–30 min, hospitalization	➀ ➁ ➂ ➃ ➄	Not reported	HAMA,MADRS,QL-Index,TCSQ,GSES
Huiqin et al. (2015)	China	+	Colorectal Cancer	–		42 vs. 42	Postoperatively, once before discharge, once in the first month after discharge and once in the third month after discharge 4 times in total	➀ ➁ ➂ ➃ ➄	Not reported	CFS, SAS, SDS
Xiaoqun et al. (2016)	China	+	Thyroid cancer	40.9 ± 6.10	39.21 ± 6.11	40 vs. 40	4 sessions of 10–15 min each during hospitalization	➀ ➁ ➂ ➃ ➄	Not reported	QLQ-C30, SAS, SDS
Jie et al. (2014)	China	+	Lung cancer, cancer of the digestive system	53.1 ± 6.9	52.1 ± 5.3	30 vs. 28	pre-chemotherapy, 3–5 sessions, 30–50 min/session	➀ ➁ ➂ ➃ ➄	1 case withdrew due to deterioration of condition, 1 case lost to visit	RPFS, SAS, SDS
Jie and Weilian (2016)	China	+	Breast cancer	33.64 ± 4.85	32.53 ± 4.75	50 vs. 50	Once a week for 40 min on Tuesdays during hospitalization	➀ ➁ ➂ ➃ ➄	Not reported	SAS;SDS,GQOL-74
Jia et al. (2023)	China	+	Breast cancer	45.6 ± 2.9	46.1 ± 2.6	52 vs. 52	Intervention management 3 months, otherwise unspecified	➀ ➁ ➂ ➃ ➄	Not reported	RPFS, FACT-B
Mei et al. (2019)	China	+	Bladder cancer	67.9 ± 6.2	66.3 ± 5.8	57 vs. 53	6 interventions over 5 months	➀ ➁ ➂ ➃ ➄	Not reported	CFS, GSES, HAMA, HAMD, QLQ-30

exp, experimental group; con, control group; PSS, Cohen’s Perceived Stress Scale; CES-D, Center Epidemiological Studies Depression Scale; CFS-C, Cancer Fatigue cale–Chinese version; QLICP-CR, Quality of Life Instruments for Colorectal Cancer Patients; SAS, Self-rating scale anxiety; SDS, Self-rating depression scale; GQOL-74, Generic Quality of Life Inventory-74; HAMA, Hamilton Anxiety Rating Scale scores; MADRS, Montgomery-Asberg Depression Rating Scale; QL-Index, Spitzer Quality of Life Index scores; Mini-MAC-19, Mini-Mental Adjustmentto Cancer Scale; QLQ-C30, European Organization for Research and Treatment of Cancer Quality of Life Questionnaire Core; BDI, Beck’s Depression Inventory; WHOQOL- BREF, WHO Quality of Life-Brief questionnaire; TCSQ, Trait Coping Style Questionnaire (TCSQ) scores; GSES, General Self-Efficacy Scale scores; SIS, Social Impact Scale; kps, Karnofsky performance status; MoCA, Montreal Cognitive Scale; HAMD, Hamilton Depression Rating Scale; FACT-B, Chicago Cancer Care Functional Rating System - Breast Cancer Survival Quality Measurement Scale; RPFS, Piper fatigue scale; RPFS, Piper Fatigue Revised Scale (Chinese version). SFBT technique: ➀ Describe the problems, ➁ Set goals, ➂ Exploring exceptions, ➃ Provide feedback, ➄ Evaluation. +: Efficiently.

### 3.2 Risk of bias

**FIGURE 2:**
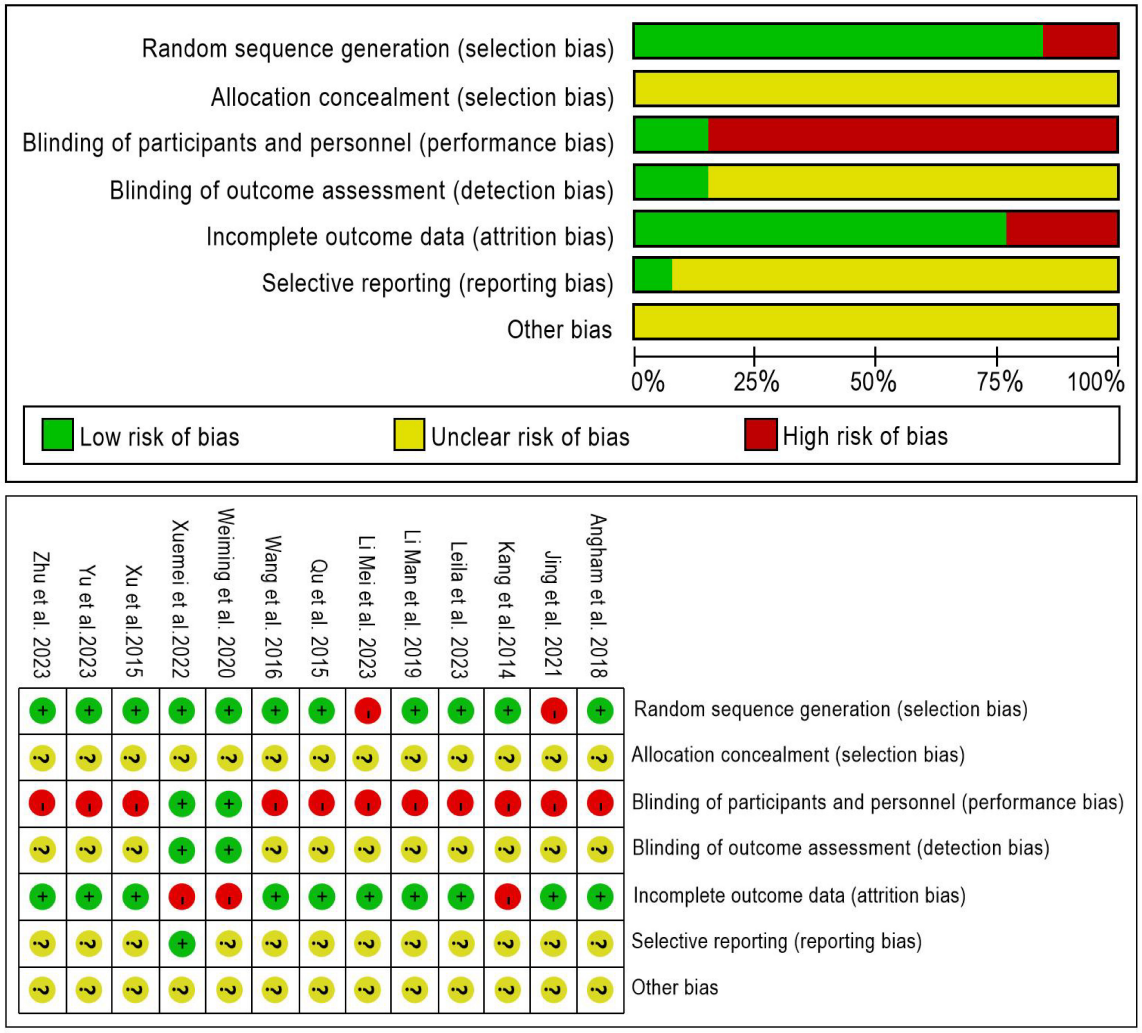
Risk of bias graph.

46% of studies (*n* = 6) reported using the random numbers method, and 38% of studies (*n* = 5) reported using other random allocation methods to assign participants to either the intervention or control group, which were considered to have a lower risk of bias. All studies did not describe allocation concealment, which is considered to have a high risk of bias. All but 2 (15%) studies did not blind participants and staff; however, blinding is more difficult to implement due to the nature of psychological interventions. Three studies (23.08%) reported lost-to-follow-up rates, ranging from 12.9∼17.5% in the intervention group and from 3.33∼19.35% in the control group. Only one (Xian et al., 2022) of the 13 studies described registration in a clinical trial registry, and none of the others did, so there may be a risk of selective reporting bias. Similarly, most studies were considered to be at unclear risk due to the presence of other biases, as there was insufficient evidence to assess the risk of these biases. Figure 2 show the risk of bias assessment. The quality of the evidence was low according to the Guidelines for Recommendation, Assessment, Development and Evaluation (see Table 3).

**TABLE 3:** Summary of evidence.

Outcomes	Illustrative comparative risks* (95% CI)		Relative effect (95% CI)	No of participants (studies)	Quality of the evidence (GRADE)	Comments
	Assumed risk	Corresponding risk				
	General intervention	SFBT intervention				
Anxiety	The mean anxiety scores ranged from -20.41 to 0.8	SMD 1.52 score lower (2.2–0.84 lower)	–	705 (8 studies)	⨁⨁◯◯low^1^,^2^,^3^	SMD -S1.52 (95% CI -2.2 to -0.84)
Depression	The mean depression scores ranged from -17.04 to 0.3	SMD 1.54 score lower(2.09–1 lower)	–	790(10 studies)	⨁⨁◯◯low^1^,^2^,^3^	SMD -1.54 (95% CI -2.09 to -1)
Fatigue	The mean fatigue scores ranged from -8.4 to 5.75	SMD 2.19 score lower (3.33–1.05 lower)	–	608(6 studies)	⨁⨁◯◯low^1^,^2^,^3^	SMD -2.19 (95% CI -3.33 to -1.05)
Quality of life	The mean quality of life scores ranged from -22.9 to 22.67	SMD 2.18 score higher (0.73–3.62 higher)	–	459(5 studies)	⨁⨁◯◯low^1^,^2^,^3^	SMD 2.18 (95% CI 0.73–3.62)

*The basis for the assumed risk (e.g., the median control group risk across studies) is provided in footnotes. The corresponding risk (and its 95% confidence interval) is based on the assumed risk in the comparison group and the relative effect of the intervention (and its 95% CI). SMD, Standardized mean difference CI, Confidence interval. GRADE Working Group grades of evidence:High quality, Further research is very unlikely to change our confidence in the estimate of effect. Moderate quality, Further research is likely to have an important impact on our confidence in the estimate of effect and may change the estimate. Low quality, Further research is very likely to have an important impact on our confidence in the estimate of effect and is likely to change the estimate. Very low quality: We are very uncertain about the estimate.

^1^Blinding aspects of most study participants and staff, as well as the risk of blinding for outcome assessment, are unclear.

^2^Inter-study heterogeneity was high.

^3^Lost visit bias, reporting bias, and other biases due to lack of registration of most clinical trials or insufficient detail.

### 3.3 Analysis of overall effects

#### 3.3.1 Effectiveness of SFBT on anxiety in cancer patients

**FIGURE 3:**
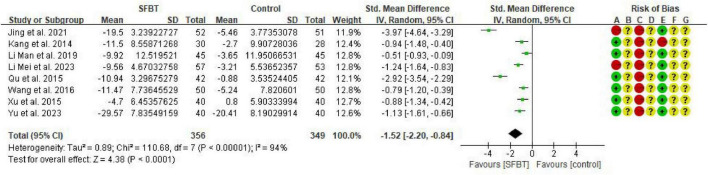
Forest plot of the effect of SFBT on anxiety in cancer patients.

Eight studies (Huiqin et al., 2015; Jie and Weilian, 2016; Jie et al., 2014; Man et al., 2019; Mei et al., 2019; Qianqian et al., 2023; Wang et al., 2021; Xiaoqun et al., 2016) were included in the analysis of anxiety, with 6 utilizing the Self-Assessment Scale for Anxiety (SAS) and 2 using the Hamilton Assessment Scale (HMAS). A random-effects model meta-analysis was conducted due to significant heterogeneity among the findings (*I*^2^ = 94%, *p* < 0.00001). The results indicated that the intervention group had a significantly better effect on improving anxiety mood compared to the control group, with a statistically significant difference (SMD = –1.52, 95% CI: –2.20 ∼ –0.84, *p* < 0.0001) (Figure 3).

#### 3.3.2 Effectiveness of SFBT on depression in cancer patients

**FIGURE 4:**
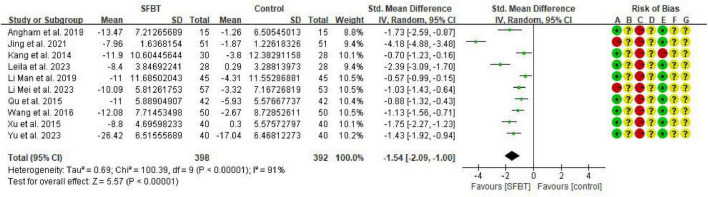
Forest plot of the effect of SFBT on depression in cancer patients.

Ten studies (Aminnasab et al., 2018; Huiqin et al., 2015; Jie and Weilian, 2016; Jie et al., 2014; Man et al., 2019; Mei et al., 2019; Pirzadi et al., 2023; Qianqian et al., 2023; Wang et al., 2021; Xiaoqun et al., 2016) reported improvement in depression. Six of the studies used the Self-rating depression scale (SDS), and the remaining four studies assessed the impact of depressed mood in cancer patients using samples of the Center Epidemiological Studies Depression Scale (CES-D), Montgomery-Asberg Depression Rating Scale (MADRS), Beck’s Depression Inventory (BDI), and Hamilton Depression Rating Scale scales (HAMD). SMD was selected for effect size combination, which showed significant heterogeneity of findings (*I*^2^ = 91%, *p* < 0.00001), and meta-analysis was performed using a random effects model. The results showed that the intervention group improved depression better than the control group with a statistically significant difference (SMD = –1.54, 95% CI: –2.09∼–1.00, *p* < 0.00001) (Figure 4).

#### 3.3.3 Effectiveness of SFBT on CRF in cancer patients

**FIGURE 5:**
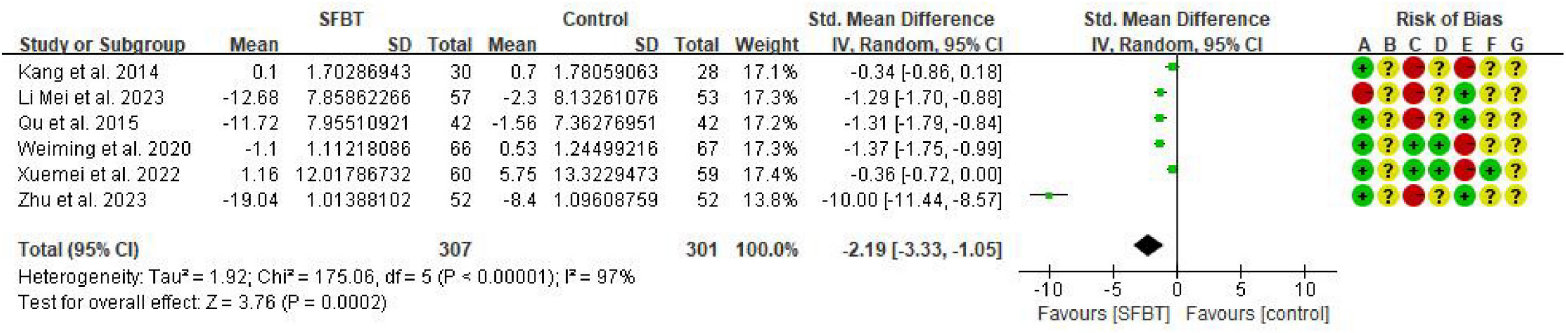
Forest plot of the effect of SFBT on cancer-related fatigue in cancer patients.

Six studies (Huiqin et al., 2015; Jia et al., 2023; Jie et al., 2014; Liu et al., 2020; Mei et al., 2019; Xian et al., 2022) report CRF outcomes in cancer patients. Three were assessed using the revised Chinese version of the Piper Fatigue Scale (CPFS), and three were assessed using the Cancer Fatigue Scale-Chinese version (CFS-C). SMD was selected for effect size combination, which showed significant heterogeneity of findings (*I*^2^ = 97%, *p* < 0.00001), and meta-analysis was performed using a random effects model. The meta-analysis showed that the SFBT intervention had a significant effect on CRF (SMD = –2.19, 95% CI: –3.33∼–1.05, *p* = 0.0002) (Figure 5).

#### 3.3.4 Effectiveness of SFBT on QoL in cancer patients

**FIGURE 6:**
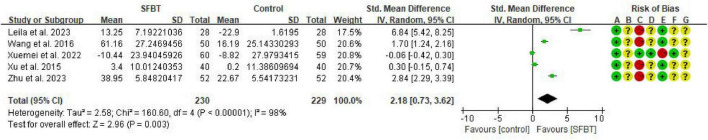
Forest plot of the effect of SFBT on quality of life in cancer patients.

Five studies (Jia et al., 2023; Jie and Weilian, 2016; Pirzadi et al., 2023; Xian et al., 2022; Xiaoqun et al., 2016) reported complete data on the impact of SFBT on QoL before and after intervention. They were assessed using different assessment scales which were the WHO Quality of Life-Brief questionnaire scale (WHOQOL-BREF), Quality of Life Questionnaire-30 (QLQ-30), the Quality of Life Instruments for Colorectal Cancer Patients scale (QLICP-CR), Chicago Cancer Care Functional Rating System-Breast Cancer Survival Quality Measurement Scale (FACT-B), and the European Organization for Research and Treatment of Cancer Quality of Life Questionnaire Core scale (EORTC QLQ-C30). The meta-analysis of 5 studies showed that SFBT-based interventions had a significant positive impact on QoL improvement (SMD = 2.18, 95% CI: 0.73∼3.62, *p* = 0.003) (Figure 6). These studies showed significant heterogeneity (*I*^2^ = 98%, *P* < 0.00001).

### 3.4 Publication bias

The funnel plot for anxiety shows a relatively symmetrical funnel, indicating little risk of publication bias.

### 3.5 Sensitivity analysis

There were no significant changes in the effect sizes and no significant directional changes in the conclusions after using different effects models and eliminating the studies one by one, suggesting that the results of this study are relatively stable and reliable.

### 3.6 Subgroup analysis

#### 3.6.1 Anxiety

##### 3.6.1.1 Subgroup analyses based on different anxiety assessment tools

There were two main anxiety assessment tools used in the included studies: the SAS (Huiqin et al., 2015; Jie and Weilian, 2016; Jie et al., 2014; Man et al., 2019; Qianqian et al., 2023; Xiaoqun et al., 2016) and the HAMA (Mei et al., 2019; Wang et al., 2021). In the subgroup assessed using the SAS, the SMD of the 6 studies combined was –1.17 (95% CI: –1.74∼–0.60, *p* < 0.0001), indicating a significant reduction in anxiety self-assessment scores with a large effect size in the SFBT group relative to the control group. In contrast, in the subgroup assessed using the HAMA, the SMD of the two studies combined was –2.59 (95% CI: –5.26∼0.09, *p* = 0.06), which was close to, but did not reach, the level of statistical significance (Supplementary Figure 1). The difference between the SAS and HAMA results suggests that the effect of SFBT was more consistent and significant on the patient self-assessment (SAS), whereas on the Hamilton Assessment scale (HAMA), although the effect size point estimate was larger, it was limited by the small number of studies (only 2), resulting in wide confidence intervals and insufficient statistical power.

##### 3.6.1.2 Subgroup analysis based on different cancer types

All subgroups of tumor types included in the analysis showed a statistically significant effect of SFBT on improving anxiety in cancer patients (Supplementary Figure 2). Although all were effective, there were significant differences in the strength of effect. Leukemia (Wang et al., 2021): SMD = -3.97 (95% CI: -4.64∼-3.29, *p* < 0.00001), Colorectal cancer (Huiqin et al., 2015): SMD = -2.92 (95% CI: -3.54 ∼ -2.29, *p* < 0.00001), Bladder cancer (Mei et al., 2019; Qianqian et al., 2023): SMD = -1.19 (95% CI: -1.50 ∼ -0.88, *p* < 0.00001), Lung cancer and cancer of the digestive system (Jie et al., 2014): SMD = -0.94 (95% CI: -1.48 ∼ -0.40, *p* = 0.0007), Breast cancer (Wang et al., 2021): SMD = -0.79 (95% CI: -1.20 ∼ -0.39, *p* = 0.0001), Cervical cancer (Man et al., 2019): SMD = -0.51 (95% CI: -0.93 ∼ -0.09, *p* = 0.02), Thyroid cancer (Xiaoqun et al., 2016): SMD = -0.88 (95% CI: -1.34 ∼ -0.42, *p* = 0.0002). Patients with tumors of the hematological system (leukemia) and digestive system (colorectal, lung cancer and cancer of the digestive system etc.) seem to benefit more from SFBT (greater absolute SMD values). Patients with breast cancer, cervical cancer, and thyroid (usually tumors with a relatively good prognosis) also benefited significantly, but with a relatively slightly smaller effect size.

##### 3.6.1.3 Subgroup analysis based on length of intervention

Subgroup analyses showed that both short-term and medium-term SFBT interventions significantly reduced anxiety levels in cancer patients (Supplementary Figure 3). Among them, there were five studies (Jie and Weilian, 2016; Man et al., 2019; Qianqian et al., 2023; Wang et al., 2021; Xiaoqun et al., 2016) for interventions completed within 3 months, with a combined SMD = -1.43 (95% CI: -2.37 ∼ -0.48, *p* = 0.003), and 3 studies (Huiqin et al., 2015; Jie et al., 2014; Mei et al., 2019) for interventions of 3–6 months, with a combined SMD = -1.68 (95% CI: -2.75 ∼ -0.61, *p* = 0.002). The results of the short-term group demonstrate that even a relatively short cycle of SFBT intervention can produce significant and large improvements in anxiety in cancer patients. The effect sizes in the medium-term group were slightly larger than those in the short-term group, but there was a large overlap between the two confidence intervals. This suggests that the effect is at least maintained and perhaps even slightly enhanced by extending the intervention to 3–6 months, but it is not yet possible to determine whether a statistically significant difference exists.

#### 3.6.2 Depression

##### 3.6.2.1 Subgroup analysis based on different depression assessment scales

Different studies have used different depression assessment scales, including the SDS, the BECK, the CES-D, the MADRS, and the HAMD, etc., The MADRS (SMD = -4.18, 95% CI: -4.88 ∼ -3.48, *p* < 0.00001) (Wang et al., 2021) and the BECK (SMD = -2.39, 95% CI: -3.09 ∼ -1.70, *p* < 0.00001) (Pirzadi et al., 2023) have shown very large effect. This suggests that the SFBT is extremely effective in the specific patient groups assessed using these scales. The SDS (SMD = -1.07, 95% CI: -1.42 ∼ -0.72, *p* < 0.00001) (Huiqin et al., 2015; Jie and Weilian, 2016; Jie et al., 2014; Man et al., 2019; Qianqian et al., 2023; Xiaoqun et al., 2016), with the largest number of included studies (*n* = 6), showed a large effect with relatively narrow confidence intervals, suggesting that this result is stable and reliable, and is a strong proxy for the overall effect. The CES-D (SMD = -1.73, 95% CI: -2.59 ∼ -0.87, *p* < 0.0001) (Aminnasab et al., 2018), and the HAMD (SMD = -1.03, CI: -1.43 ∼ -0.64, *p* < 0.00001) (Mei et al., 2019) also showed moderate to large effects. Subgroup analyses revealed that although there were significant differences in effect sizes, the SFBT demonstrated a significant reduction in depressive symptoms regardless of which depression assessment scale was used (Supplementary Figure 4).

##### 3.6.2.2 Subgroup analysis based on different cancer types

Among the various types of cancer patients analyzed, SFBT showed a significant reduction in depressive symptoms. Among them, the absolute value of SMD was the largest in leukemia patients (*n* = 1, SMD = -4.18, 95% CI: -4.88 ∼ -3.48, *p* < 0.00001) (Wang et al., 2021), suggesting that SFBT was particularly effective in alleviating depression in leukemia patients; SMD was also relatively high in patients with thyroid (*n* = 1, SMD = -1.75, 95% CI: -2.27 ∼ -1.23, *p* < 0.00001) (Xiaoqun et al., 2016) and breast cancers (*n* = 3, SMD = -1.72, 95% CI: -2.53 ∼ -0.91, *p* < 0.0001) (Aminnasab et al., 2018; Jie and Weilian, 2016; Pirzadi et al., 2023), suggesting that SFBT had a significant effect in these groups. The absolute values of SMD for patients with bladder cancer (*n* = 2, SMD = -1.20, 95% CI: -1.59 ∼ -0.82, *p* < 0.00001) (Mei et al., 2019; Qianqian et al., 2023), colorectal cancer (*n* = 1, SMD = -0.88, 95% CI: -1.32 ∼ -0.43, *p* = 0.0001) (Huiqin et al., 2015), lung cancer and cancer of the digestive system (*n* = 1, SMD = -0.70, 95% CI: -1.23 ∼ -0.16, *p* = 0.01) (Jie et al., 2014) and cervical cancer (*n* = 1, SMD = -0.57, 95% CI: -1.23 ∼ -0.16, *p* = 0.008) (Man et al., 2019) decreased sequentially, but all of them reached statistical significance (Supplementary Figure 5).

##### 3.6.2.3 Subgroup analysis based on length of intervention

The results showed that the combined effect size SMD = -1.86 (95% CI: -2.64 ∼ -1.08, *p* < 0.00001) (Aminnasab et al., 2018; Jie and Weilian, 2016; Man et al., 2019; Pirzadi et al., 2023; Qianqian et al., 2023; Wang et al., 2021; Xiaoqun et al., 2016) was significantly higher in the short-term group (*n* = 7) than in the medium-term group (*n* = 3, SMD = -0.90, 95% CI: -1.16 ∼ -0.64, *p* < 0.00001) (Huiqin et al., 2015; Jie et al., 2014; Mei et al., 2019). This implies that shorter sessions of SFBT may result in greater improvement in depressive symptoms (Supplementary Figure 6).

##### 3.6.2.4 Subgroup analysis based on different study countries

Differences in cultural and medical settings may affect the implementation and effectiveness of SFBT. We conducted subgroup analyses by the country in which the study was conducted, and the results showed that the combined effect size SMD = -2.11 (95% CI: -2.76 ∼ -1.47 *p* < 0.00001) for the Iranian studies (*n* = 2) (Aminnasab et al., 2018; Pirzadi et al., 2023), which was slightly higher than that of the Chinese studies (*n* = 8, SMD = -1.43, 95% CI: -2.03 ∼ -0.82 *p* < 0.00001) (Huiqin et al., 2015; Jie and Weilian, 2016; Jie et al., 2014; Man et al., 2019; Mei et al., 2019; Qianqian et al., 2023; Wang et al., 2021; Xiaoqun et al., 2016). This suggests that the ameliorative effect of SFBT on depression in cancer patients may be more significant in the Iranian cultural context (Supplementary Figure 7).

#### 3.6.3 CRF

##### 3.6.3.1 Subgroup analysis based on assessment scales

The effect of SFBT on improving CRF in cancer patients reached statistically significant levels in studies using different scales, but the effect sizes varied widely. Three studies used the PFS (Huiqin et al., 2015; Mei et al., 2019; Xian et al., 2022), which had a large and significant effect size (SMD = -3.77, 95% CI: -6.83 ∼ -0.72, *p* = 0.02). The other three articles (Jia et al., 2023; Jie et al., 2014; Liu et al., 2020) used the CFS, which had a large and highly significant effect size (SMD = -0.98, 95% CI: -1.63 ∼ -0.32, *p* = 0.003) (Jia et al., 2023; Jie et al., 2014; Liu et al., 2020). The confidence intervals contained negative values but did not cross zero indicating robust results. This suggests that SFBT has a clear and significant effect on the improvement of CRF symptoms assessed using CFS, and that the effect sizes are within the expected range of reasonableness (Supplementary Figure 8).

##### 3.6.3.2 Subgroup analyses based on cancer type

Differences in effect sizes and significance were found across subgroups of cancer types. Among them, the bladder cancer subgroup (*n* = 1) showed a significant amelioration of CRF by SFBT intervention with an SMD = -1.29 (95% CI: -1.70 ∼ -0.88), *p* < 0.00001) (Mei et al., 2019), a significant effect and narrow confidence interval, and a very robust result. The combined SMD for the colorectal cancer subgroup (*n* = 2) was -0.82 (95% CI: -1.76 ∼ 0.11, *p* = 0.08) (Huiqin et al., 2015; Xian et al., 2022), which was close to significant. The lower limit of the confidence interval (-1.76) showed a large potential for improvement, but the upper limit (0.11) spanned zero, suggesting that SFBT may have a tendency to alleviate CRF in patients with colorectal cancer, but the uncertainty of the results is large due to the limited number of studies. The combined SMD for the breast cancer subgroup (*n* = 2) was -5.66 (95% CI: -14.12 ∼ 2.80, *p* = 0.19) (Jia et al., 2023; Liu et al., 2020), which did not reach significance and the confidence interval crossed the null line, suggesting that the evidence is not yet sufficient to support a clear efficacy of SFBT on CRF in breast cancer patients. The SMD for the thyroid cancer subgroup (*n* = 1) was -0.34 (95% CI: -0.86 ∼ 0.18, *p* = 0.20) (Jie et al., 2014), again not showing a significant effect (Supplementary Figure 9).

##### 3.6.3.3 Subgroup analysis based on length of intervention

Both short-term and medium-term SFBT interventions significantly reduced CRF levels in cancer patients. This provides a rationale for flexible setting of the intervention period in clinical practice. Among them, the short-term subgroup included 1 study with an SMD = -1.37 (95% CI: -1.75 ∼ -0.99, *p* < 0.00001) (Liu et al., 2020). The medium-term subgroup contained 5 studies with a combined SMD = -2.43 (95% CI: -3.90 ∼ -0.96, *p* = 0.001) (Huiqin et al., 2015; Jia et al., 2023; Jie et al., 2014; Mei et al., 2019; Xian et al., 2022), again reaching a highly significant level. It is worth noting that the absolute value of the effect size was larger in medium-term subgroup than in the short-term subgroup, but the confidence intervals were wider in the former due to the inclusion of more studies and possible heterogeneity. It is not possible to directly conclude that “the longer the duration, the better the effect,” but only suggests that SFBT may have a more sustained or cumulative effect on CRF over a longer intervention period of 3–6 months (Supplementary Figure 10).

#### 3.6.4 Quality of life

##### 3.6.4.1 Subgroup analysis based on different QoL assessment scales

Subgroup analyses demonstrated the effect of SFBT on QoL improvement, and the results measured using different scales of assessment were highly variable. The WHOQOL-BREF had a large and highly significant effect size (SMD = 6.84, 95% CI: 5.42∼8.25, *p* < 0.00001) (Pirzadi et al., 2023). The FACT-B is a cancer-specific scale, especially for breast cancer, with a large and highly significant effect size (SMD = 2.84, 95% CI: 2.29∼3.39, *p* < 0.00001) (Jia et al., 2023). The results showed that SFBT was highly effective in improving core QoL dimensions in breast cancer patients. The effect size of the GQOL-74 was large and highly significant (SMD = 1.70, 95% CI: 1.24∼2.16, *p* < 0.00001) (Jie and Weilian, 2016). It indicates that SFBT has a significant positive impact on patients’ self-reported overall QoL. The confidence interval for the QLQ-C30 spanned zero (SMD = 0.30, 95% CI: -0.15 ∼ 0.74, *p* = 0.19) (Xiaoqun et al., 2016), indicating that the effect of SFBT did not reach a statistically significant level in this study. The QLICP-CR confidence intervals were narrow and tightly centered around zero (SMD = -0.86, 95% CI: -0.42 ∼ 0.30, *p* = 0.74) (Xian et al., 2022), suggesting that the SFBT did not produce an observable effect on QOL assessed using this scale in this study of bowel cancer patients (Supplementary Figure 11).

##### 3.6.4.2 Subgroup analysis based on different cancer types

Three studies were included in the breast cancer subgroup in this analysis, whereas only one study each was included in the bowel and thyroid cancer subgroups. The subgroup analysis revealed the most significant intervention effect of SFBT in breast cancer patients (SMD = 3.64, 95% CI: 1.72 ∼ 5.55, *p* = 0.0002) (Jia et al., 2023; Jie and Weilian, 2016; Pirzadi et al., 2023), indicating that SFBT significantly improved the QoL of breast cancer patients. In contrast, the results for the subgroups of colorectal cancer (SMD = -0.06, 95% CI: -0.42 ∼ 0.30, *p* = 0.74) (Xian et al., 2022) and thyroid cancer (SMD = 0.30, 95% CI: -0.15 ∼ 0.74, *p* = 0.19) (Xiaoqun et al., 2016) patients did not show a significant effect of SFBT. This suggests that SFBT as a psychological intervention may have a limited impact on QoL in these groups (Supplementary Figure 12).

##### 3.6.4.3 Subgroup analysis based on length of intervention

The effect of SFBT was significant and the effect size was large in short-term (SMD = 2.79, 95% CI: 0.57∼5.02, *p* = 0.01) (Jie and Weilian, 2016; Pirzadi et al., 2023; Xiaoqun et al., 2016). Suggests that a relatively short course of SFBT (usually within a few weeks to 3 months) can have a significant positive impact on QOL in cancer patients. The SMD for the medium-term was 1.38, 95% CI = -1.46 to 4.22, *p* = 0.34 (Jia et al., 2023; Xian et al., 2022), with a moderate but not significant effect size. Confidence intervals are wide and span zero, indicating that the effect is unstable and does not reach a statistically significant level in this time frame (Supplementary Figure 13).

## 4 Discussion

This meta-analysis provides a quantitative synthesis of the effects of SFBT on psychological and physical symptoms in cancer patients. While the overall findings suggest beneficial effects, a critical appraisal reveals nuances and important limitations that must be considered when interpreting these results.

Our analysis indicates that SFBT demonstrates a significant beneficial effect on anxiety symptoms. The therapy’s solution-oriented, future-focused approach likely helps patients reframe threats and enhance coping self-efficacy, thereby reducing the cognitive and emotional burden of anxiety (Hankin et al., 2023; Shi et al., 2024). However, the observed effect was not uniform across assessment tools. The significant reduction measured by the self-reported SAS, contrasted with the non-significant effect on the clinician-rated HAMA, raises critical questions. This discrepancy may indicate that SFBT primarily alleviates the subjective experience of anxiety, which is effectively captured by self-report scales, while its impact on the objective somatic and behavioral manifestations assessed by the HAMA may be more limited (von Känel et al., 2021). This is a crucial distinction for clinical practice, suggesting SFBT may be most appropriate for patients whose distress is primarily cognitive-emotional. The variation in effect sizes across cancer types (e.g., strong effects in leukemia) further suggests that the intervention’s efficacy is context-dependent, potentially mediated by baseline distress levels and disease-specific stressors (Gray et al., 2021). Nonetheless, the small number of studies for most cancer subtypes precludes definitive conclusions regarding its specificity. It is noteworthy that extremely high heterogeneity existed across studies (*I*^2^ = 94%), indicating substantial variation in the extent to which SFBT affects anxiety. Although our subgroup analyses explored differences by assessment tool and cancer type, significant unexplained variability remains. Factors not captured in our analysis, such as baseline anxiety severity, cancer stage, or concurrent pharmacological treatments, may be important contributors to this heterogeneity. Therefore, the pooled SMD of -1.52 represents an average of disparate effects, and its generalizability to specific clinical populations should be viewed with caution.

Similarly, SFBT appears effective in reducing depressive symptoms, with large effect sizes observed for specific scales like the MADRS and BDI. This aligns with the theory that SFBT’s emphasis on personal strengths, goal-setting, and small successes can break the cycle of helplessness and negative cognition that characterizes depression (Harshita and Rubina, 2022). The finding that short-term interventions were slightly more effective than medium-term ones is particularly intriguing. It may support SFBT’s core strength as a rapid-change modality, capitalizing on early “novelty” effects and providing timely intervention to prevent the entrenchment of depressive patterns (Calear and Christensen, 2010; Fawzy et al., 2001). However, this finding must be interpreted with caution, as it could also be influenced by differences in patient populations or study methodologies between the short- and medium-term groups. It is noteworthy that cancer treatment is a dynamic process. As the disease progresses, treatment side effects accumulate, and long-term psychological adaptation burdens mount, depressive symptoms may fluctuate or worsen over time. Consequently, as a short-term intervention, the initial effects of SFBT may diminish over time, particularly when confronting persistent disease-related stressors. Future research should incorporate longer-term follow-up assessments to evaluate the sustainability of SFBT’s effects and its applicability across different stages of the disease. The cultural consistency of effects (e.g., in China and Iran) underscores its cross-cultural applicability (Żak and Pȩkala, 2024), yet the overall evidence remains marred by heterogeneity in scale usage and patient characteristics. Furthermore, the substantial heterogeneity observed across these studies (*I*^2^ = 91%) warrants careful consideration. While our subgroup analyses provided some explanatory insights, a significant amount of unexplained variability remains. This suggests that the efficacy of SFBT for depressive symptoms is not uniform and may be moderated by factors beyond our analytical scope. For instance, biological subtypes of depression, the presence of coexisting psychosocial stressors, or cultural adaptations of SFBT protocols may all influence outcomes. Therefore, the pooled standardized mean difference (SMD) of -1.54 should be interpreted as a summary of heterogeneous effects rather than a precise, universally applicable estimate. This heterogeneity underscores the importance of individualized clinical judgment when considering SFBT implementation in cancer care.

The positive effect of SFBT on CRF is a significant finding, pointing to its role in addressing a pervasive and debilitating physical symptom. The proposed mechanism involves psychological intervention improving stress management, potentially regulating the HPA axis and enhancing prefrontal modulation of fatigue-related brain networks (Alvarado-García et al., 2025). By fostering cognitive restructuring and behavioral activation, SFBT may help patients break the “fatigue-avoidance” cycle and gradually rebuild activity tolerance (Yuen and Cunningham, 2014). However, the results across tumor types were inconsistent. The dramatic effect in bladder cancer patients may be linked to SFBT’s efficacy in addressing body image-related shame, a potent source of psychological fatigue. In contrast, the non-significant results for breast and thyroid cancer patients highlight that the pathophysiology of CRF and responsiveness to psychological interventions may vary substantially across cancers. The extremely large but heterogeneous effect size for the PFS scale, compared to the more robust effect for the CFS, further suggests that the choice of measurement instrument critically influences the observed outcomes. This heterogeneity calls into question the generalizability of the CRF findings and indicates a need for more standardized assessment in future research. The remarkably high heterogeneity (*I*^2^ = 97%) for CRF outcomes necessitates a guarded interpretation of the large pooled effect size (SMD = -2.19). The divergence in findings based on assessment scale (PFS vs. CFS) and the inconsistent effects across cancer types suggest that the efficacy of SFBT for CRF is highly context-dependent. Unmeasured variables, including treatment phase (e.g., active chemotherapy vs. survivorship) and biological mechanisms of fatigue specific to different cancers, likely play a crucial role. This heterogeneity limits the certainty with which a unified conclusion can be drawn.

Finally, our results confirm that SFBT can enhance the QoL of cancer patients, likely through its synergistic effect on reducing both psychological and physical symptoms. A key mechanistic insight from our analysis pertains to the domain of action for SFBT. The subgroup analysis based on different QoL assessment tools revealed a critical pattern: SFBT’s positive impact was most pronounced on scales emphasizing broad psychosocial wellbeing and subjective life satisfaction (e.g., WHOQOL-BREF, FACT-B, GQOL-74), whereas it was negligible or non-significant on scales primarily focused on physical symptom burden and functional status (e.g., QLQ-C30, QLICP-CR). This pattern strongly suggests that SFBT’s primary action mechanism is psychosocial, and its benefits are most directly reflected in patients’ subjective perception of their quality of life and psychosocial adaptation, rather than in a direct amelioration of core physical symptoms. This is a vital distinction for clinicians and researchers when selecting outcome measures and setting realistic expectations for SFBT interventions. The lack of a significant effect for longer-term (3–6 months) interventions on QoL is surprising and warrants further investigation; it may be due to the small number of studies, the influence of external factors over time, or a potential “ceiling effect” of the brief therapy model.

### 4.1 Limitations

This study provides valuable insights through a systematic review and meta-analysis of the efficacy of SFBT on anxiety, depression, CRF, and quality of life QoL in cancer patients. However, several limitations remain. The most significant limitation lies in the overall low certainty of evidence for all outcomes. This primarily stems from significant methodological flaws in the included randomized controlled trials (RCTs), such as the widespread absence of allocation concealment and blinding, as well as the lack of trial registration in most studies. These deficiencies introduced a high risk of bias, substantially undermining the robustness of the study’s conclusions. Although the pooled effect size was statistically significant, its interpretation requires caution due to the inherent fragility of the original evidence.

Furthermore, although the search covered multiple databases, the included studies predominantly originated from Asia (particularly China and Iran), which may limit the cross-cultural generalizability of the conclusions. The number of available studies, participants, and the mix of Asian and Western populations were limited. This reduces the precision of our effect estimates. Results need validation in more RCTs. Studies varied in sample characteristics, intervention protocols, and outcome measures. While subgroup analyses explored some sources of heterogeneity, unexplained heterogeneity may persist. Future research could employ stricter criteria or sensitivity analyses for further investigation.

Regarding intervention implementation, SFBT was predominantly delivered by nurses rather than licensed mental health professionals in the studies included in this analysis. While reflecting clinical practice, this may introduce implementation heterogeneity (e.g., variations in skill proficiency) and compromise intervention consistency. Future research comparing the efficacy of professional versus non-professional practitioners could advance the standardization of intervention protocols.

This study primarily included published RCTs. Although attempts were made to construct funnel plots, the small number of studies made it difficult to reliably detect publication bias. Therefore, more high-quality research is still needed to confirm the true efficacy of SFBT. Furthermore, this analysis primarily focused on short-term outcomes, with a significant gap in the absence of long-term follow-up data. Given that the psychological state of cancer patients often fluctuates with disease progression, future research should evaluate whether the efficacy of SFBT is sustained over the long term and whether enhanced interventions are required to maintain these effects.

The findings of this study, based on the integration of quantitative data, did not delve into the qualitative aspects of SFBT implementation (such as patient experiences and key therapeutic factors). Additionally, this study exhibits high heterogeneity, potentially attributable to the diversity of included studies—including cancer types, diagnostic stages, and patient baseline characteristics. Although we conducted subgroup analyses (e.g., cancer type and intervention duration), most original studies failed to report cancer staging or diagnostic details, limiting further analysis. Future research should standardize reporting of such variables to reduce heterogeneity. Finally, future studies may integrate qualitative research methods to provide a more comprehensive evaluation. It is recommended to conduct larger-scale, high-quality RCTs covering diverse cancer types and stages to validate the long-term efficacy and generalizability of SFBT. Additionally, exploring integrated models combining SFBT with other interventions could better address the complex psychological needs of cancer patients.

### 4.2 Implications for clinical practice

The results of this meta-analysis have important implications for the practice of psychosocial support for cancer patients. The study confirmed the significant effect of SFBT in improving psychological symptoms such as anxiety and depression in cancer patients, which provides an evidence-based basis for the clinical introduction of this therapy. Healthcare organizations may consider incorporating SFBT into daily care as a useful supplement to existing interventions. In particular, the short-course nature of SFBT makes it a highly effective option when resources are limited or when patients are unable to receive long-term treatment. The results of the subgroup analysis suggest that SFBT may be suitable for different types of cancer patients, but the strategy can be adjusted according to the specific conditions of the patients. In addition, considering the impact of the intervention duration on the effect, the clinical work can adopt the mode of “short intensive + regular follow-up” to consolidate the effect of the treatment and prevent the recurrence of symptoms. Psychological problems such as anxiety and depression not only affect the quality of life of patients, but also may have a negative impact on treatment adherence and physical recovery. Therefore, clinics should pay more attention to the psychological status of cancer patients and routinely conduct psychological screening to identify patients in need of timely intervention. For patients with significant anxiety or depression, psychotherapeutic services should be actively provided in addition to necessary pharmacological treatments. SFBT, as a non-pharmacological, patient-centered intervention, can be safely administered concurrently with antitumor therapy to help patients better cope with the psychological challenges of the treatment process. This integration of SFBT principles into holistic care can help create a positive therapeutic atmosphere and enhance patients’ psychological resilience.

## 5 Conclusion

This study evaluated the effectiveness of SFBT in cancer patients by Meta-analytic system, and the results showed that SFBT could significantly improve the patients’ anxiety and depression symptoms, and reduce CRF and improve the QoL to some extent. Subgroup analyses showed that the efficacy of SFBT was consistent across assessment scales, cancer types, and intervention durations, but the effect sizes may be influenced by these factors. Overall, SFBT demonstrated encouraging results as a short-course, positively oriented psychological intervention for psychosocial support in cancer. However, due to the overall poor quality of the evidence, we cannot draw definitive conclusions and therefore caution is still needed in applying the current evidence. Therefore, when considering the application of SFBT in clinical practice, an individualized assessment must be conducted based on the patient’s specific disease stage, psychosocial background, symptom presentation, and treatment goals. In the future, more rigorous studies could be conducted to validate not only the effectiveness of SFBT interventions, but, more importantly, to examine which assessment modalities are more sensitive to the corresponding cancer outcomes and the length of the intervention that achieves the best therapeutic outcome for the patient.

## Data Availability

The original contributions presented in this study are included in this article/Supplementary material, further inquiries can be directed to the corresponding authors.
