# Supplementary material for: Effectiveness of solution-focused brief therapy on cancer patients: a systematic review and meta-analysis
Source: Front Psychol. 2026 Feb 12;17:1741088. doi: 10.3389/fpsyg.2026.1741088 (PMC12936997; doi:10.3389/fpsyg.2026.1741088)
Supplement: Supplementary file 1 [file Table_1.docx]

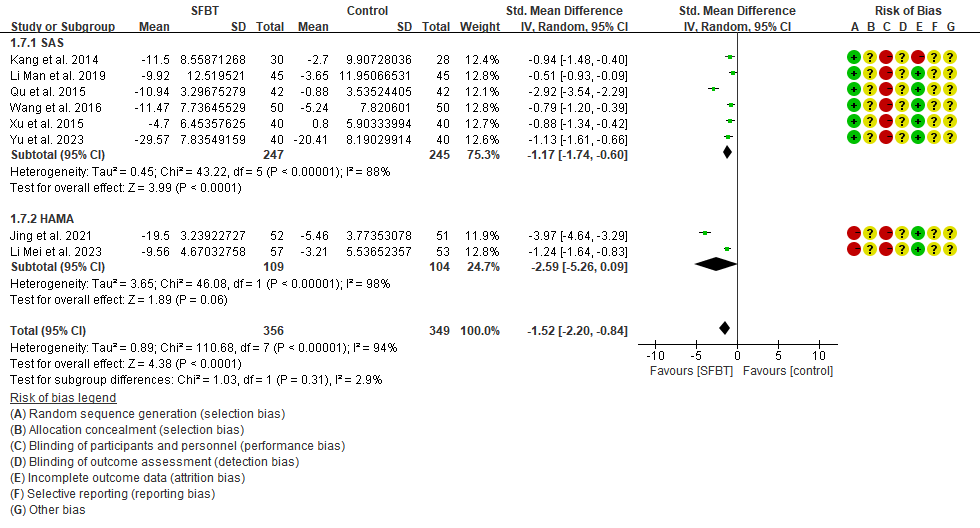


Supplementary figure 1. Results of subgroup analysis of anxiety based on assessment tools


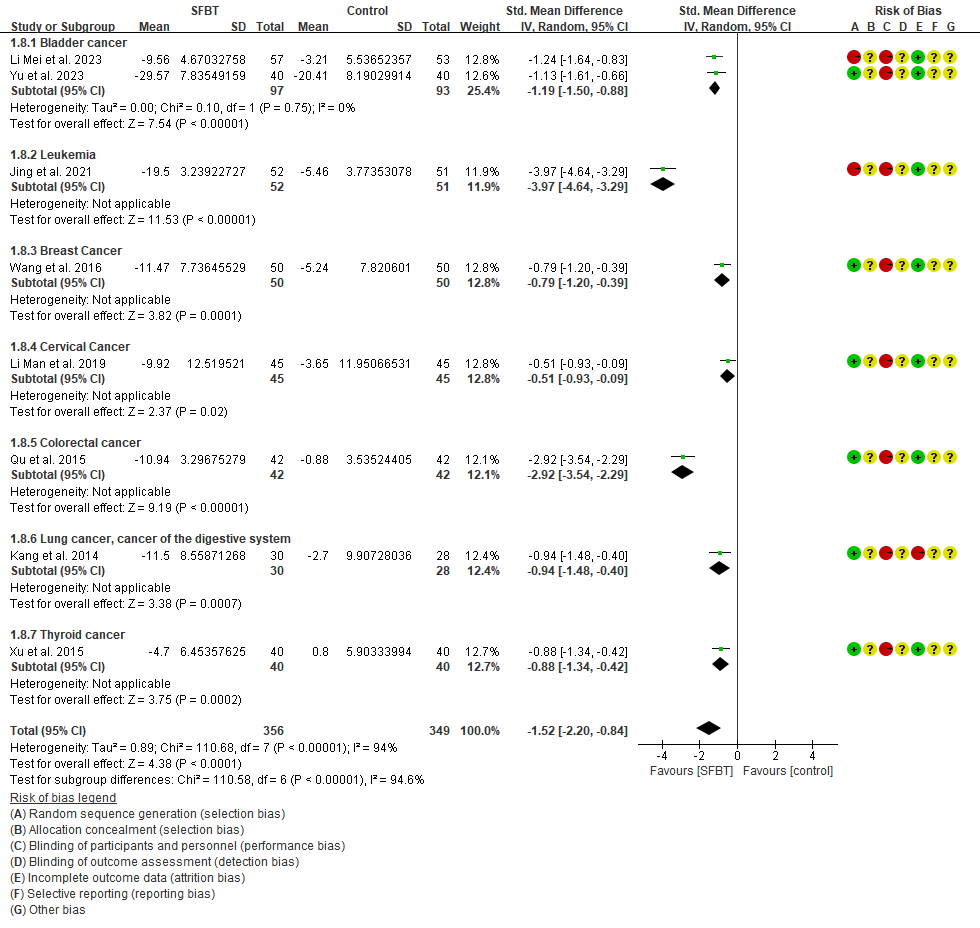


Supplementary figure2 Results of subgroup analysis of anxiety based on cancer type


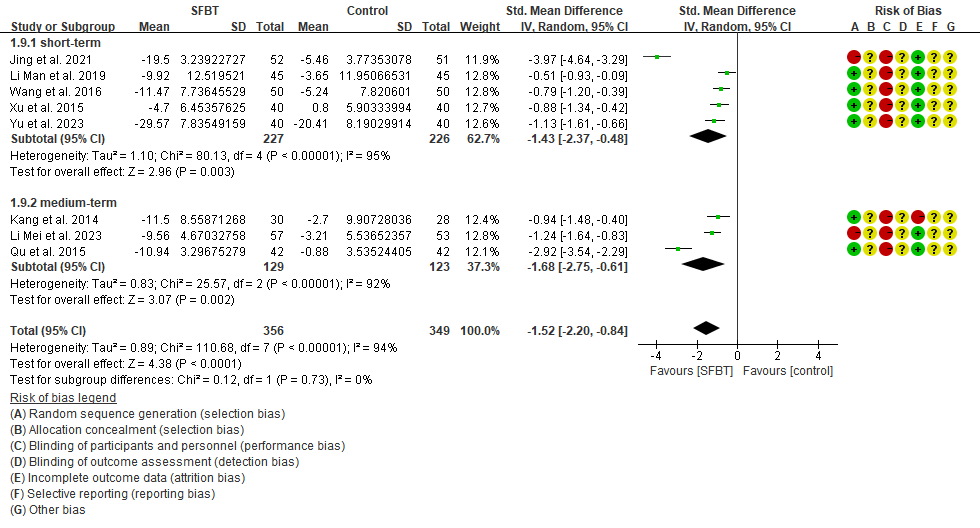


Supplementary figure 3 Results of subgroup analysis of anxiety based on length of intervention


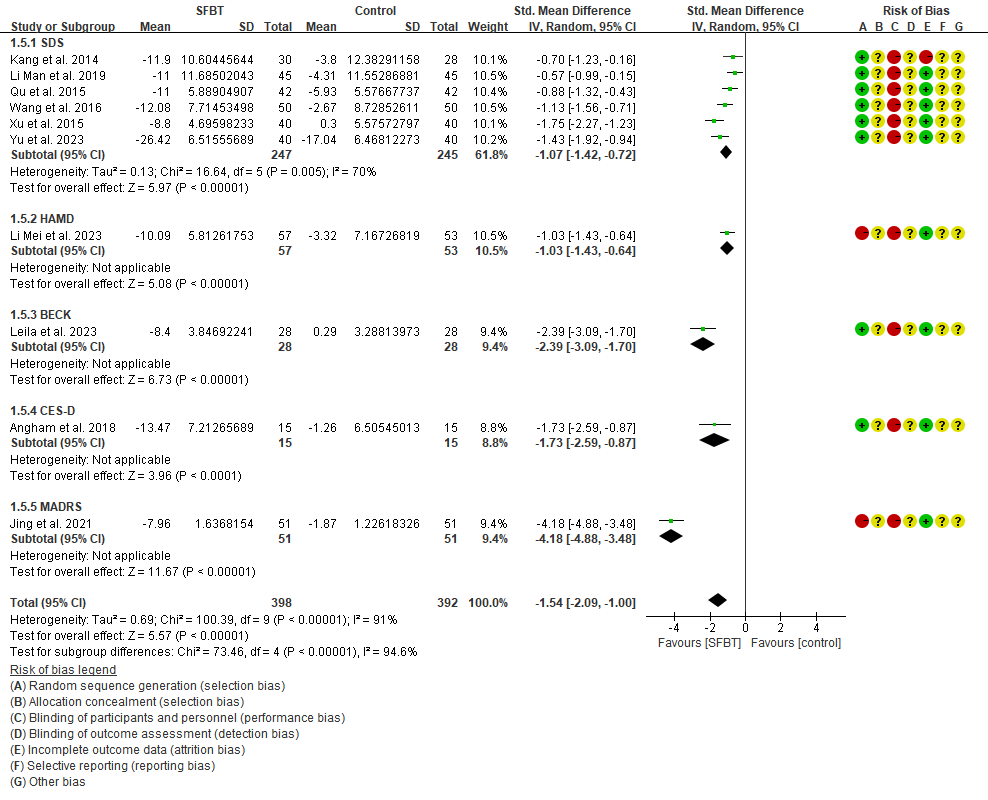


Supplementary figure 4 Results of subgroup analysis of depression based on assessment scales


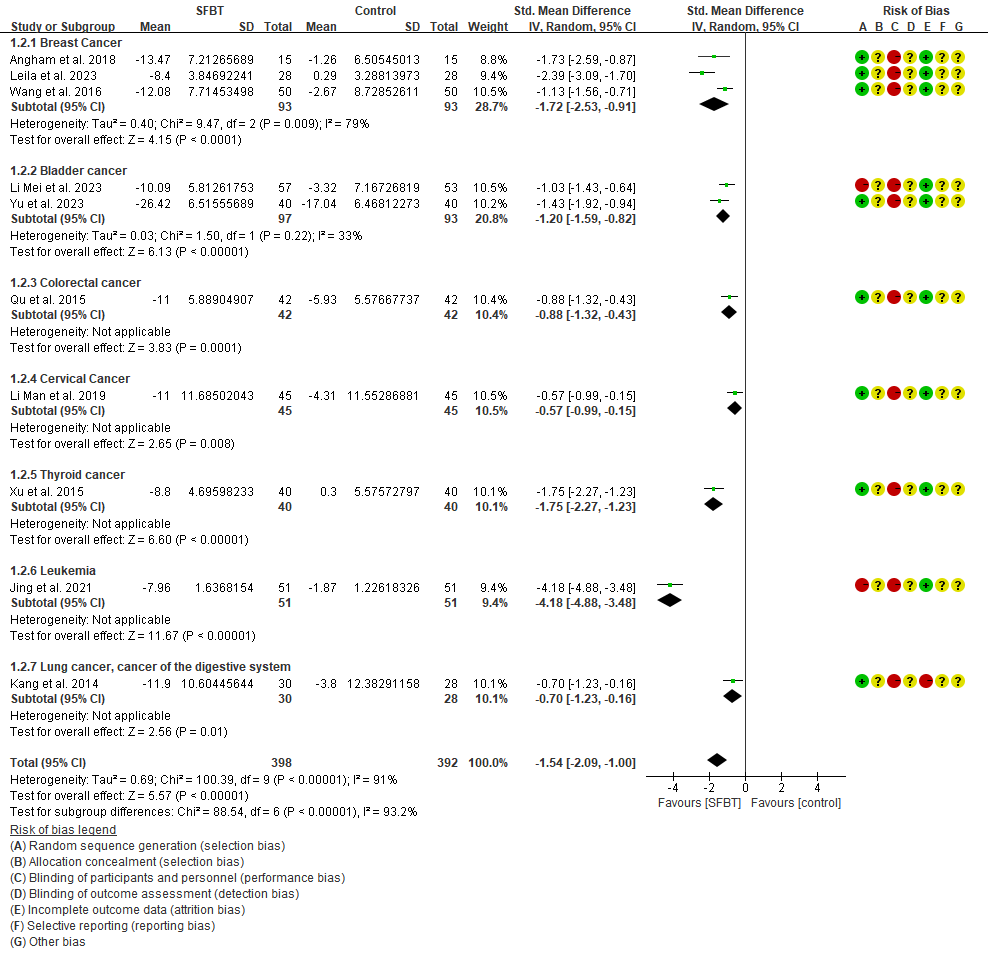


Supplementary figure 5 Results of subgroup analysis of depression based on cancer type


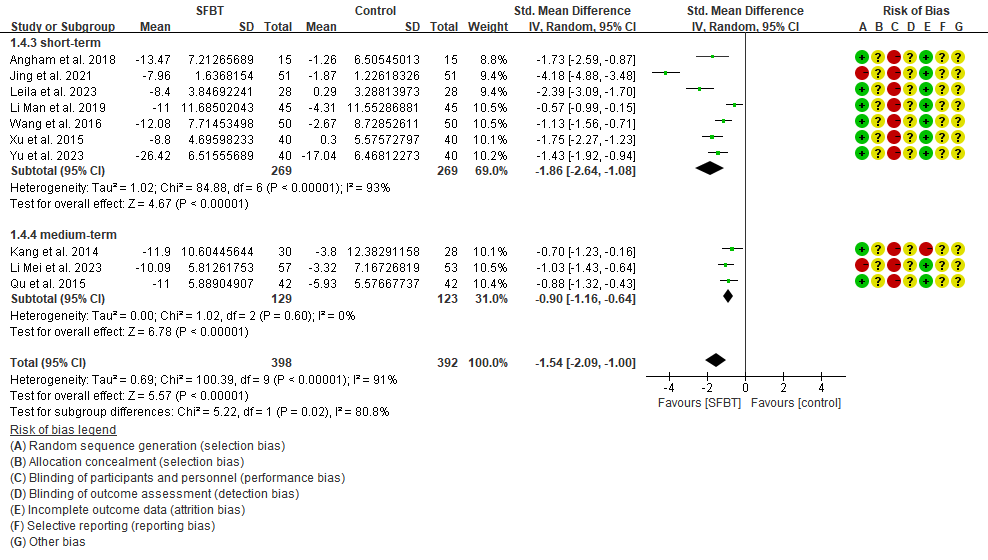


Supplementary figure 6 Results of subgroup analysis of depression based on intervention time


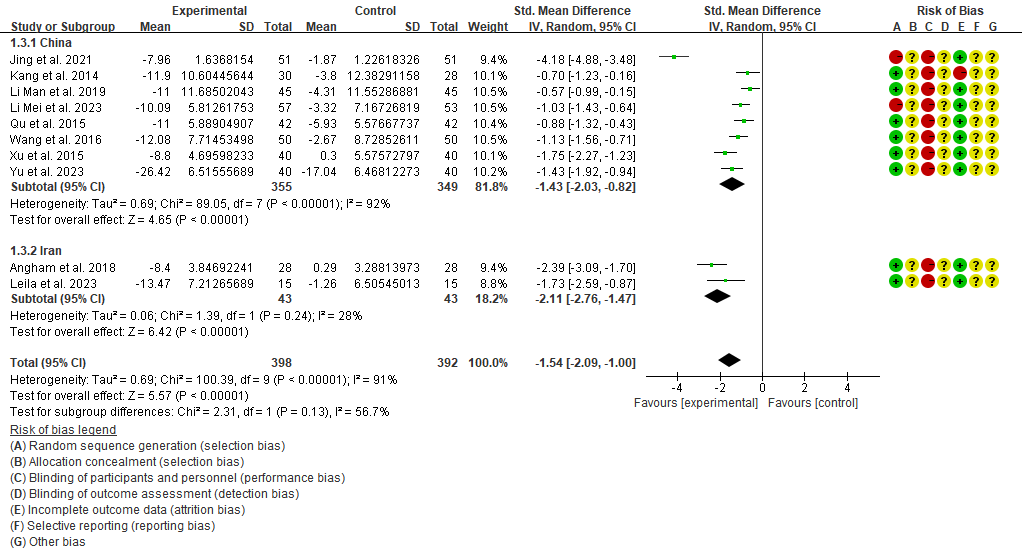


Supplementary figure 7 Results of subgroup analysis of depression based on different countries


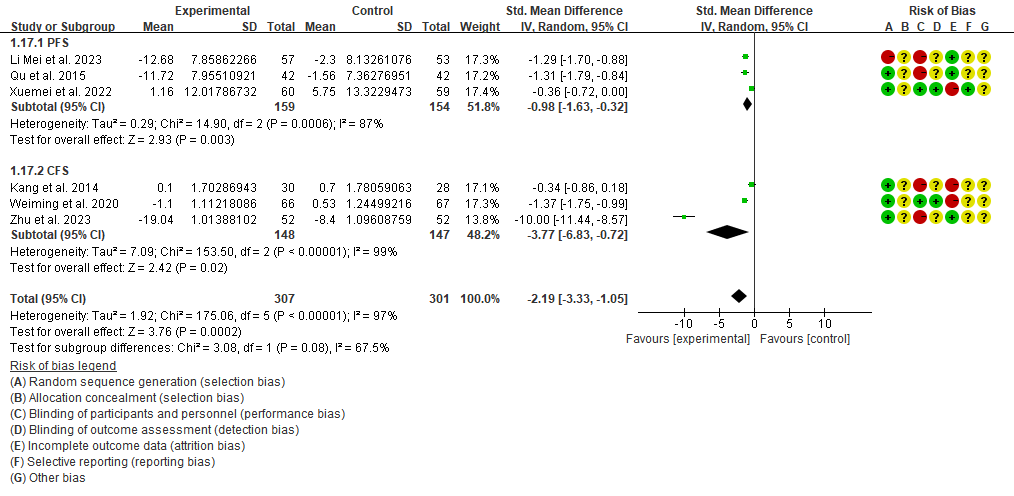


Supplementary figure 8 Results of subgroup analysis of fatigue based on assessment scales


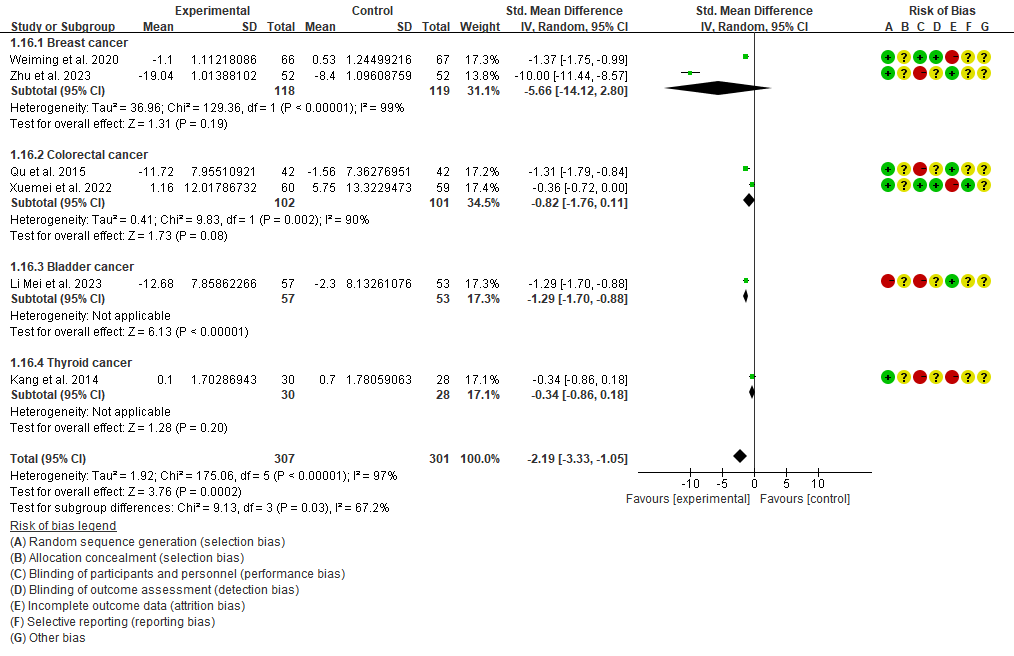


Supplementary figure 9 Results of subgroup analysis of fatigue based on cancer type


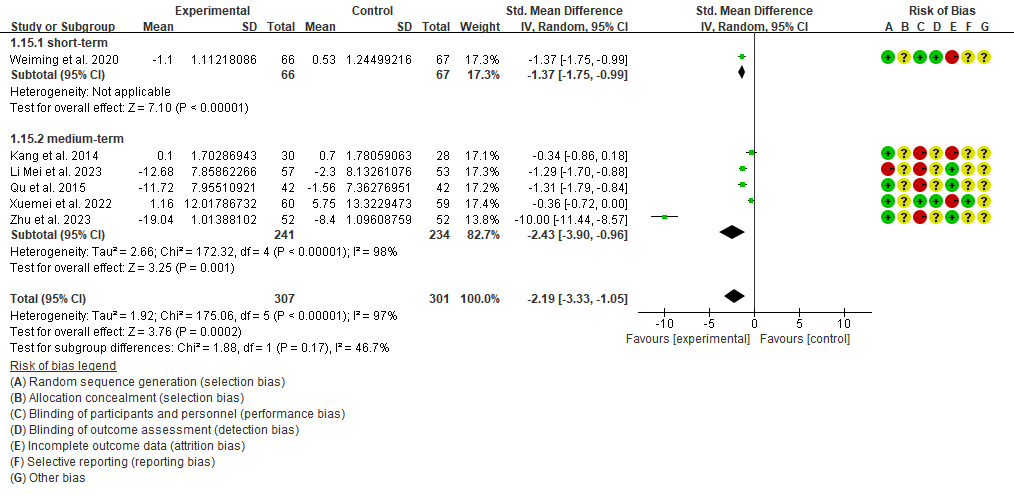


Supplementary figure 10 Results of subgroup analysis of fatigue based on intervention time


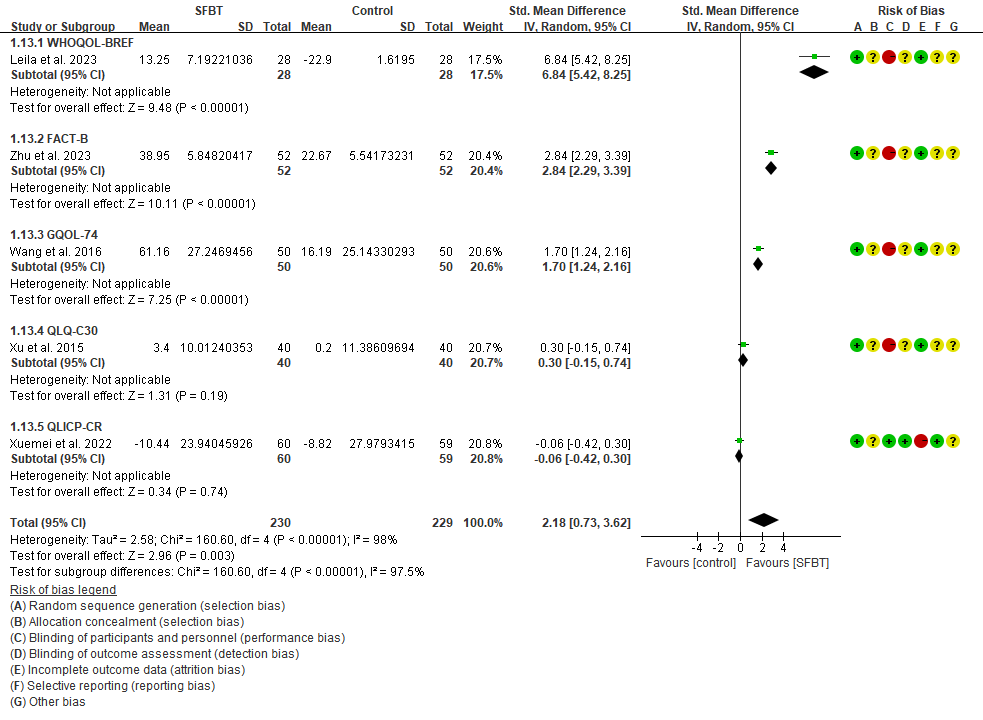


Supplementary figure 11 Results of subgroup analysis of quality of life based on assessment scales


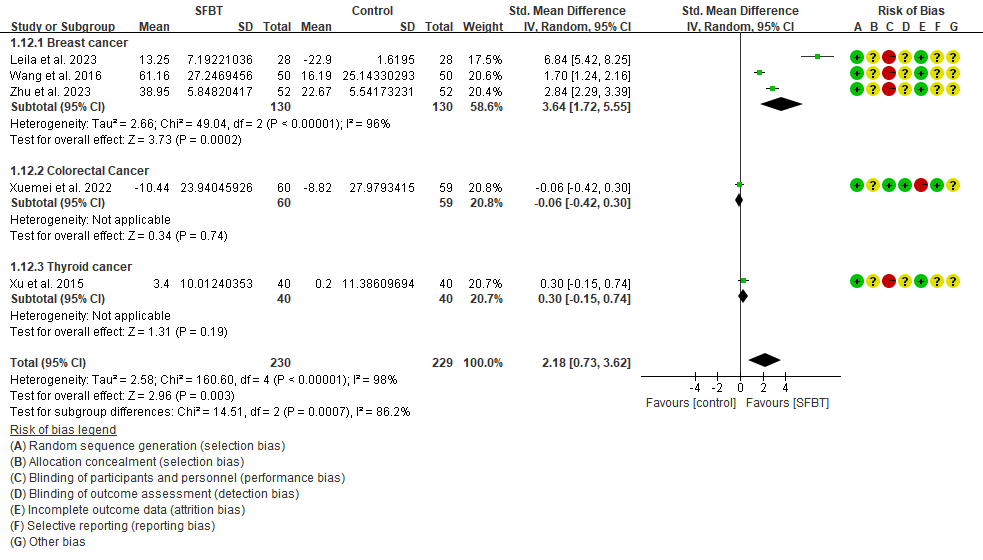


Supplementary figure 12 Results of subgroup analysis of quality of life based on cancer type


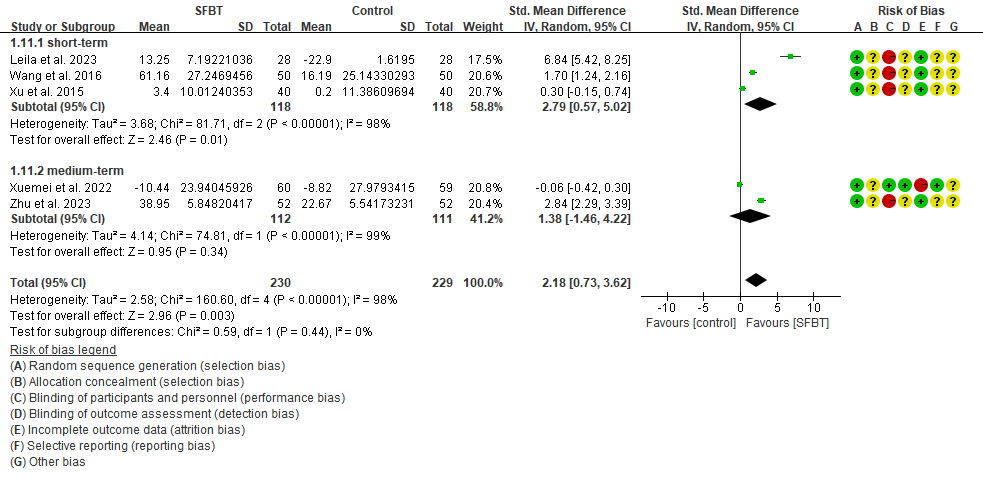


Supplementary figure 13 Results of subgroup analysis of quality of life based on intervention time
